# AlphaFold and Docking Approaches for Antibody–Antigen and Other Targets: Insights From CAPRI Rounds 47–55

**DOI:** 10.1002/prot.26801

**Published:** 2025-01-20

**Authors:** Ragul Gowthaman, Minjae Park, Rui Yin, Johnathan D. Guest, Brian G. Pierce

**Affiliations:** ^1^ University of Maryland Institute for Bioscience and Biotechnology Research Rockville Maryland USA; ^2^ Department of Cell Biology and Molecular Genetics University of Maryland College Park Maryland USA

**Keywords:** AlphaFold, antibody, CAPRI, docking, recognition, ZDOCK, ZRANK

## Abstract

Accurate modeling of the structures of protein–protein complexes and other biomolecular interactions represents a longstanding and important challenge for computational biology. The Critical Assessment of PRedicted Interactions (CAPRI) experiment has served for over two decades as a key means to assess and compare current approaches and methods through blind predictive scenarios, highlighting useful strategies, and new developments. Here we describe the performance of our laboratory's team in recent CAPRI rounds, which included submissions for 10 modeling targets. Our team utilized a range of docking and modeling approaches, including ZDOCK, Rosetta, and ZRANK, to model, refine, and score protein–protein and protein–DNA complexes. For recent targets we utilized adaptations of AlphaFold to generate models, leading to near‐native models for an antibody–peptide target, and a highly accurate (but low ranked) model for an antibody–MHC complex. These results underscore the utility of AlphaFold‐based protocols for predictive protein complex modeling, including for immune recognition, and highlight considerations regarding the use of AlphaFold confidence metrics in model selection.

## 1 Introduction

Protein complexes are critical for biological function, and high resolution structures of those interactions can provide major insights, while enabling structure‐based design to modify or inhibit those interactions [1, 2]. As experimental structural determination is time‐consuming, costly, and not always successful, accurate predictive computational methods to model structures of protein–protein and other complexes would be immensely useful. To this end, many modeling approaches have been developed over several decades, including protein–protein docking methods, as reviewed previously [3, 4, 5], in addition to template‐based interaction modeling methods [5, 6, 7]. While new docking and modeling approaches that are developed are often assessed using docking benchmarks [8, 9, 10], testing of approaches in blind predictive challenges like the Critical Assessment of Predicted Interactions (CAPRI) [11] can give a highly useful indication of relative and expected modeling accuracy in real‐world scenarios.

We have participated in CAPRI as team member [12, 13, 14, 15, 16] and a predictor team [17, 18, 19] in numerous CAPRI and CASP‐CAPRI rounds, starting in CAPRI Round 3. In recent CAPRI rounds, the availability of AlphaFold and related end‐to‐end deep learning methods that can “fold‐and‐dock” protein complexes [20] has dramatically improved the capability of the community to model targets [19], including those with large binding conformational changes that formerly posed challenges to traditional rigid‐body or semi‐rigid docking protocols [9]. Here we describe our performance in CAPRI rounds 47–55, with an emphasis on recent targets that provided useful lessons on AlphaFold, its adaptations and limitations, and its capability to model immune recognition.

## 2 Results

### 2.1 Overview of Protocols and Performance

As summarized in Table 1, we utilized integrative approaches, employing a range of modeling, refinement, and scoring algorithms, to generate models for 10 targets in these CAPRI rounds. Of note, for Round 49 (Target 163) we did not submit predictions, and our Round 50 and Round 54 CASP‐CAPRI performance is described separately [18, 19] and is not included here. For Rounds 47, 48, and 52 our protocols included modeling in ZDOCK 3.0.2 [22] and M‐ZDOCK [25], refinement with Rosetta FastRelax [32] and scoring with the REF15 function [38] by Rosetta InterfaceAnalyzer [37], as well as rescoring by ZRANK2 [35], in addition to other complementary modeling and scoring techniques. Unfortunately, no successful near‐native models were submitted for those difficult targets. For Round 53, Target 187 had no successful models generated by our team, but the same complex with provided bound component DNA structure (Target 188) resulted in an acceptable accuracy complex model, which was generated by ZDOCK with DNA parameters [14, 39] with refinement and scoring using Rosetta FastRelax [32] and ZRANK [34].

**TABLE 1:** Modeling approaches and performance for CAPRI 47–55 targets.

Round	Target	Type	Difficulty^a^	Modeling^b^	Refinement/scoring^c^	Accuracy^d^
47	160	Domain organization, nanobody‐antigen	Difficult	RosettaAntibody, ZDOCK	FCC, RosettaFR, IRAD, ZRANK2	0
48	161	Toxin homodimer	Difficult	Modeller, M‐ZDOCK, SymDock	RosettaFT, RosettaFR, RosettaIA, ZRANK2	0
	162	Enzyme‐inhibitor	Difficult	M‐ZDOCK, ZDOCK	FCC, RosettaFR, ZRANK, ZRANK2	0
52	186	Protein–protein, loop modeling	Difficult	SWISS‐MODEL, RosettaNGK	ZRANK2	0
53	187	Protein–DNA	Difficult	AlphaFold2, HDOCK	RosettaFR	0
	188	Protein–DNA	Difficult	ZDOCK	RosettaFR, ZRANK	1*
55	231	Antibody–peptide	Difficult	AlphaFold2ms, Modeller	pep‐pLDDT	4**/1*
	232	Protein–protein	Easy	AlphaFold2ms, Modeller	RosettaFR, RosettaIA, model confidence	2**
	233	Antibody–antigen	Difficult	AlphaFold2ms, ZDOCK, Modeller	FCC, I‐pLDDT, RosettaFR, ZRANK2	0
	234	Antibody–antigen	Difficult	AlphaFold2ms, ZDOCK, Modeller	FCC, I‐pLDDT, RosettaFR, ZRANK2	0

^a^ Target difficulty, as noted by CAPRI organizers.

^b^ Modeling approach(es) used for target, including RosettaAntibody [21], ZDOCK [22], Modeller [23], Rosetta SymDock (SymDock) [24], M‐ZDOCK [25], SWISS‐MODEL [26], Rosetta next generation kinematic closure (RosettaNGK) [27], AlphaFold2 [20, 28], HDOCK [29], and AlphaFold2 with massive sampling adapted from Wallner [30] (AlphaFold2ms).

^c^ Refinement and scoring approaches used for target, including fraction of common contacts (FCC) [31], Rosetta FastRelax (RosettaFR) [32], IRAD [33], ZRANK [34], ZRANK2 [35], Rosetta FloppyTail (RosettaFT) [36], Rosetta InterfaceAnalyzer (RosettaIA) [37], AlphaFold2 average peptide residue pLDDT score (pep‐pLDDT) [28], AlphaFold2 interface residue average pLDDT score (I‐pLDDT) [28], AlphaFold2 model confidence (model confidence) [20].

^d^ Number of acceptable (*) or medium (**) accuracy models among the five top assessed models for the target.

For Round 55, we utilized AlphaFold2 in addition to other modeling and docking approaches for the four targets (Table 1). Based on its effectiveness in the recent CASP‐CAPRI round [19] and in our benchmarking [40], we employed massive sampling in AlphaFold adapted from the approach of Wallner [30], pooling sets of predictions generated by AlphaFold v2.1, 2.2, and 2.3 with dropout enabled, with and without templates. This corresponded to 11 sets of parameters (Table 2), for which equal numbers of models were generated for each target complex; for instance, 25 models per parameter set were used to generate the 275 total models for Target 231. While minor target‐specific strategy modifications were employed for each target, as noted below, no antibody‐specific strategies were used.

**TABLE 2:** Massive sampling parameters used for Round 55 targets.

AlphaFold model	Templates	# recycles
2.1	No	3
2.1	Yes	3
2.1	No	21
2.1	Yes	21
2.2	No	3
2.2	Yes	3
2.2	No	9
2.2	No	21
2.2	Yes	21
2.3	No	20^a^
2.3	Yes	20^a^

^a^ Default value for maximum recycles for AlphaFold 2.3.

Our strategy and performance for Targets T231, 233, and 234 are described separately below.

### 2.2 Target 231: M2 Antibody‐FLAG Peptide Complex

For Target 231 (M2 Fab in complex with FLAG peptide) we utilized AlphaFold with v2.1–2.3 massive sampling, as noted above, generating 275 models in total for the target. In accordance with our recent modeling [40] and for computational efficiency, we included only the antibody variable domains for AlphaFold modeling, adding antibody constant domains prior to model submission using Modeller. For model ranking and selection, we used average AlphaFold pLDDT confidence scores of the peptide residues (peptide pLDDT), in order to have an interface‐focused confidence metric and based on indications that interface pLDDT is a useful metric for selecting antibody–antigen models [40]. The five models for this target were near‐native, with four medium and one acceptable CAPRI accuracy models (Table 1). The top‐ranked submitted model, shown in comparison with the experimentally determined complex structure [41] in Figure 1, had medium CAPRI accuracy, with interface root‐mean‐square distance (RMSD) 0.96 Å from the X‐ray structure and accurate peptide backbone and side chain positioning. Its confidence scores were in the general range of accurate antibody–antigen models [40], with 0.9 model confidence score and 81.6 peptide pLDDT score. Analysis of the larger set of our team's 100 submitted models (Figure 2A–C) shows that peptide pLDDT appears to be more effective at discriminating accurate models for this target versus AlphaFold2 model confidence and ZRANK2 scores.

**FIGURE 1:**
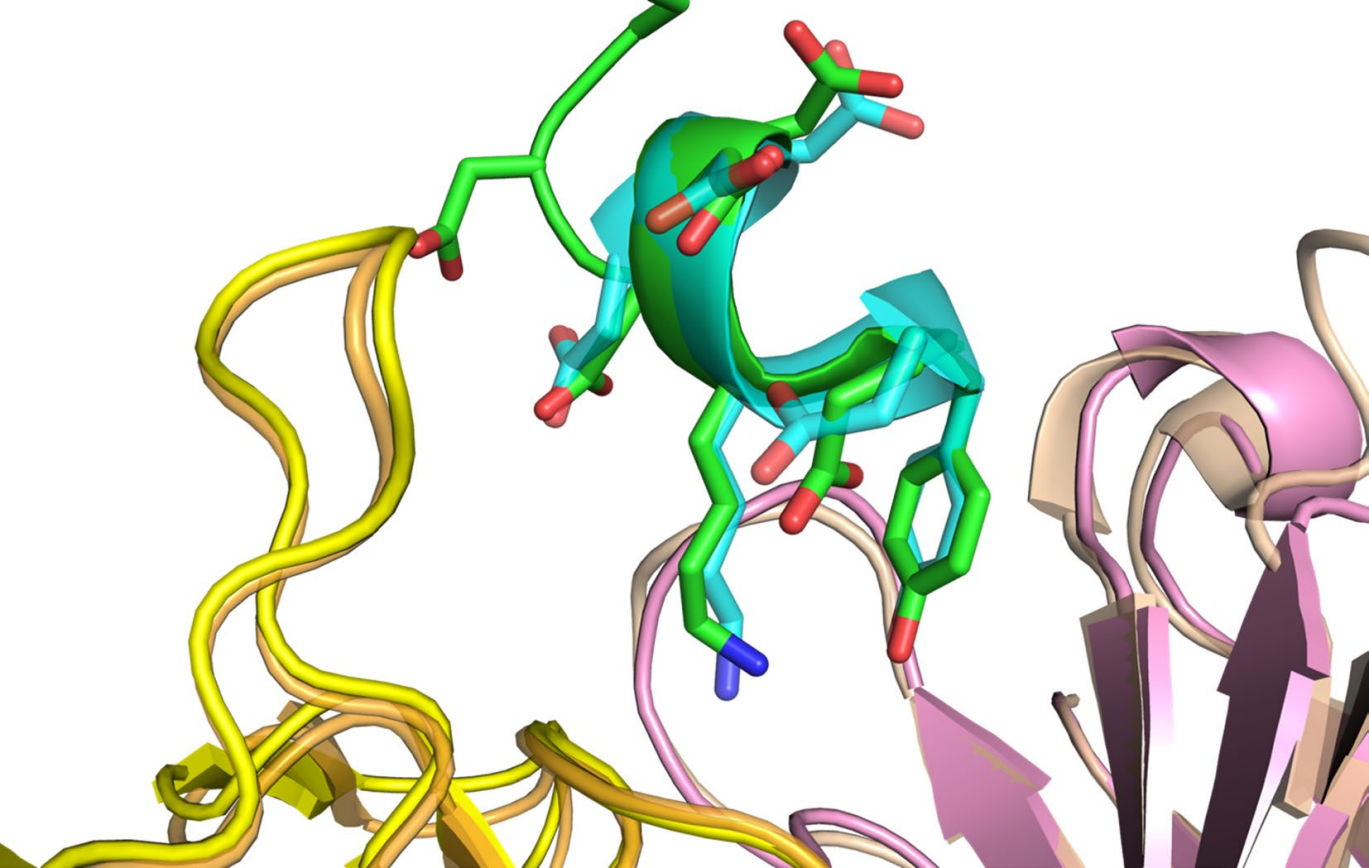
Model and X‐ray structure of Target 231 antibody‐peptide complex. The modeled complex, shown in green (FLAG peptide), pink (M2 antibody heavy chain), and yellow (M2 antibody light chain) is shown superposed by antibody variable domain onto the experimentally determined structure of the complex (PDB code 8RMO) [41], which has chains colored cyan (FLAG peptide), tan (M2 antibody heavy chain), and orange (M2 antibody light chain). Modeled and experimentally determined peptide side chains are shown as sticks. The model shown is the top‐ranked model submitted by our team, which has 0.90 fraction of native contacts (fnat), 0.96 Å interface RMSD from the experimentally determined structure, and medium CAPRI accuracy.

**FIGURE 2:**
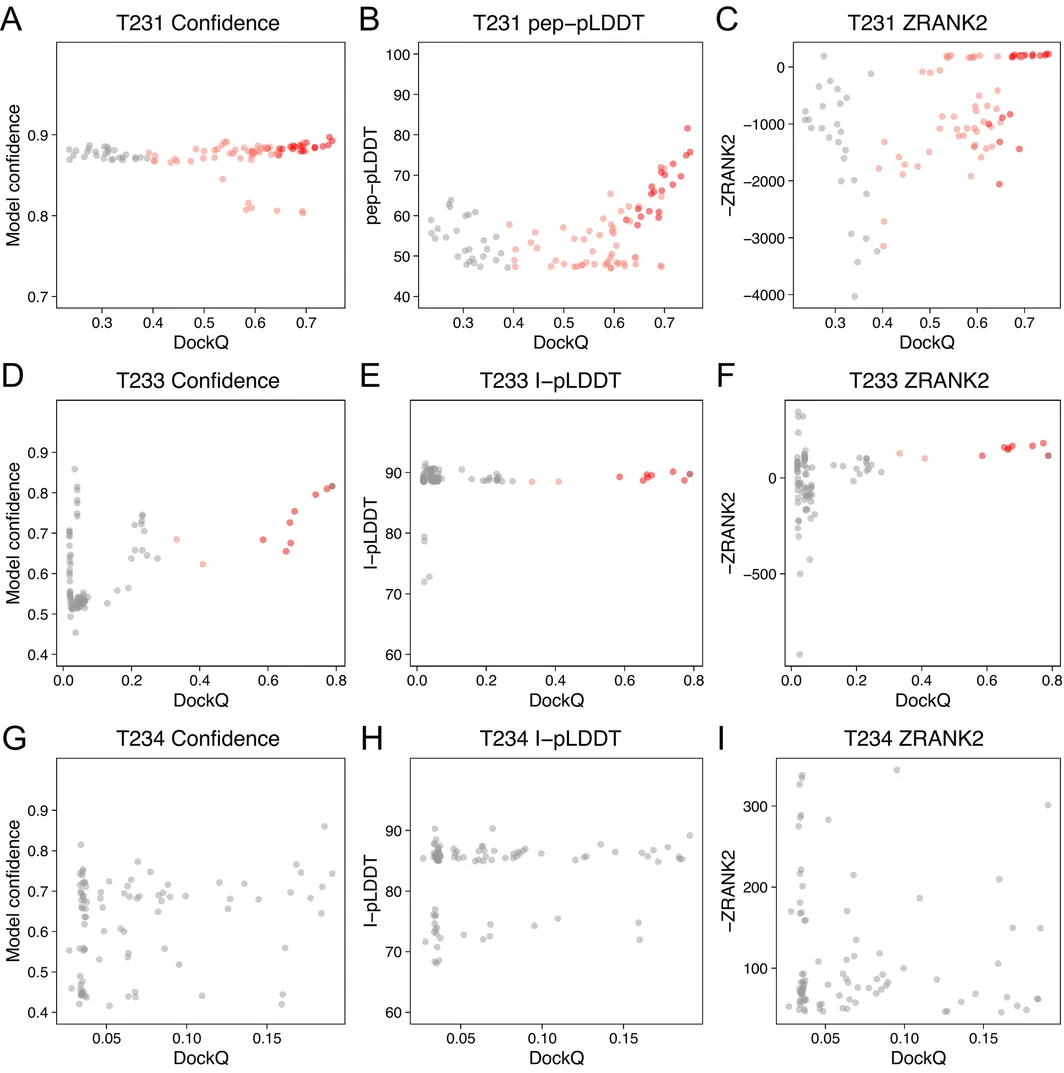
Scores versus accuracies for Target 231, 232, and 234 models. Scores versus accuracies for (A–C) Target T231 (T231), (D–F) Target 233 (T233), and (G–I) Target 234 (T234), for the 100 submitted models per target. Scores used are (A, D, G) AlphaFold2 model confidence score, (B) peptide pLDDT, (E, H) interface pLDDT, and (C, F, I) ZRANK2 score, with ZRANK2 score negated to facilitate comparison with the other scores. Accuracies are represented by DockQ score [42], which represents model accuracy on a scale from 0 to 1, and points are colored by CAPRI accuracy level (gray: incorrect, pink: acceptable, red: medium, dark red: high). A small number of the 100 submitted models from T233 (*N* = 8) and T234 (*N* = 8) were generated by ZDOCK and thus do not have AlphaFold2 confidence or pLDDT scores available, so are not shown.

### 2.3 Target 232: Phosphatase Protein Complex

For Target 232, we generated models using Modeller and an identified homologous protein complex (PDB code 5W0W) as template, in addition to AlphaFold with massive sampling which was used to generate 275 total models. The template‐based models were refined using Rosetta FastRelax [32], followed by interface scoring with the Rosetta REF15 function [38] in Rosetta InterfaceAnalyzer [37]. After manual inspection of top‐ranked homology models from Rosetta score and AlphaFold model confidence, we selected three models from template‐based modeling and two from AlphaFold for the top five. While the template‐based models were incorrect, the two AlphaFold massive sampling models, which were originally produced by AlphaFold v2.2, were near‐native with medium CAPRI accuracy.

### 2.4 Target 233 and Target 234: Antibody–MHC Complexes

Targets 233 and 234 were other cases of antibody recognition, with antibodies engaging an HLA‐A*11:01 MHC‐β2m complex and an HLA‐A*02:01 MHC‐β2m complex, respectively. As noted in Table 1, we employed both AlphaFold2 with massive sampling (1100 pooled models per complex) as well as ZDOCK to model the complexes, with the latter employed due to the relatively low confidence scores observed in preliminary AlphaFold 2.3 models. Models from ZDOCK and AlphaFold2 were then clustered using fraction of common contacts (FCC), and ranked by ZRANK2 [35] and AlphaFold interface residue pLDDT (I‐pLDDT) scores (the latter for AlphaFold2‐generated models only), with I‐pLDDT using 4.0 Å residue distance cutoff as used previously [40]. Prior to scoring, ZDOCK models were refined using the Rosetta FastRelax protocol [32]. The five top submitted models for each target, which included top‐scoring models from ZDOCK and AlphaFold2, did not include accurate models.

Interestingly, based on CAPRI assessment of our 100 submitted models for the Target 233 scoring set, our team submitted a high accuracy model for that target (interface RMSD = 0.94 Å, ligand RMSD = 3.26 Å). This is to our knowledge the only model of that accuracy level submitted by any team for either Target 233 or 234, indicating that AlphaFold2 and massive sampling can generate accurate models for at least one of those challenging interfaces. That model was generated by AlphaFold v2.1, supporting the use of v2.1–2.3 pooling versus 2.3 alone. We investigated the scores from our Target 233 set (Figure 2D–F), and while the I‐pLDDT score of that model was not has high as less accurate submitted models (89.8, ranked #24 of AlphaFold2 models), it had a relatively high model confidence score of 0.816 (ranked #2 of AlphaFold2 models). Additionally, while some false positive models are present with relatively high model confidence scores (> 0.7), there is an overall association between model confidence and model accuracy (DockQ), whereas for I‐pLDDT and ZRANK2 such associations are not apparent. For Target 234 submissions, we likewise compared scores with model quality (Figure 2G–I), and although some confidence scores are relatively high (> 0.7), all models were incorrect with poor DockQ scores (< 0.2), indicating that model confidence score can be limited as an absolute metric of success. This retrospective analysis indicates that model confidence would have been a more effective ranking score than I‐pLDDT score for Target 233, suggesting that model confidence, which is a combination of AlphaFold's interface pTM and pTM scores [20], should likely be considered as a primary metric for prospective ranking and model assessment. Additionally, interface pTM score that is focused on the relevant protein–protein interface being modeled (not output by default in AlphaFold2, but possible to obtain) would help to avoid the bias from other protein interfaces in a complex that are reflected in default model confidence score, as observed for our Target 231 models (Figure 2A).

## 3 Discussion

We utilized a combination of deep learning in AlphaFold2 and established docking and modeling approaches for recent CAPRI targets, demonstrating the capability of AlphaFold2 and massive sampling to model even challenging antibody–antigen targets in blind predictive scenarios. However, there still seems to be room for improvement, as supported by benchmarking that indicates that approximately half of antibody–antigen cases do not have near‐native models with AlphaFold2 and massive sampling [40]. The recently released AlphaFold3 [43] is reported by its authors to show some improvement in antibody–antigen modeling accuracy versus AlphaFold2 [43], but it still seems to have an upper limit of performance in that study, and separate benchmarking and use in blind prediction challenges like CAPRI are needed to confirm its relative and overall utility.

For model scoring, AlphaFold‐based local interface confidence score (I‐pLDDT) has previously shown utility in absolute and relative scoring of antibody–antigen and protein complex model quality [40, 44]. However, we found with at least one target (Target 233) that AlphaFold's default model confidence scores can be better indicators of accuracy. The limitations of AlphaFold‐based confidence scores in identification of accurate models suggest that adaptations of those scores, or non‐AlphaFold scores which would better enable pooling with non‐AlphaFold models, would be worth pursuing as a means of accurate model identification. One example in this regard is from the Venclovas team, which had relatively high success in a recent CASP‐CAPRI round with a non‐AlphaFold model scoring function [19, 45].

The recent CAPRI rounds have demonstrated that even with major advances in complex modeling accuracy, challenges remain. As with previous CAPRI rounds, the successes and limitations from these rounds point to avenues for improvement, driving advances and innovations that can then be tested in upcoming CAPRI and CASP‐CAPRI experiments.

## Author Contributions

**Ragul Gowthaman:** conceptualization, investigation, writing – review and editing, writing – original draft, formal analysis. **Minjae Park:** conceptualization, investigation, writing – review and editing, writing – original draft, formal analysis. **Rui Yin:** conceptualization, investigation, writing – review and editing, formal analysis. **Johnathan D. Guest:** conceptualization, investigation, writing – review and editing, formal analysis. **Brian G. Pierce:** writing – original draft, writing – review and editing, conceptualization, investigation, funding acquisition, formal analysis.

## Data Availability

The data that support the findings of this study are available from the corresponding author upon reasonable request.

## References

[1] L. Cao , I. Goreshnik , B. Coventry , et al., “De Novo Design of Picomolar SARS‐CoV‐2 Miniprotein Inhibitors,” Science 370, no. 6515 (2020): 426–431. 10.1126/science.abd9909 PMC7857403 32907861

[2] R. U. Kadam , J. Juraszek , B. Brandenburg , et al., “Potent Peptidic Fusion Inhibitors of Influenza Virus,” Science 358, no. 6362 (2017): 496–502. 10.1126/science.aan0516 PMC5659926 28971971

[3] I. Halperin , B. Ma , H. Wolfson , and R. Nussinov , “Principles of Docking: An Overview of Search Algorithms and a Guide to Scoring Functions,” Proteins 47, no. 4 (2002): 409–443. 10.1002/prot.10115 12001221

[4] I. A. Vakser , “Challenges in Protein Docking,” Current Opinion in Structural Biology 64 (2020): 160–165. 10.1016/j.sbi.2020.07.001 PMC7666046 32836051

[5] K. A. Porter , I. Desta , D. Kozakov , and S. Vajda , “What Method to Use for Protein‐Protein Docking?,” Current Opinion in Structural Biology 55 (2019): 1–7. 10.1016/j.sbi.2018.12.010 PMC6669123 30711743

[6] P. J. Kundrotas , Z. Zhu , J. Janin , and I. A. Vakser , “Templates Are Available to Model Nearly all Complexes of Structurally Characterized Proteins,” Proceedings of the National Academy of Sciences of the United States of America 109, no. 24 (2012): 9438–9441. 10.1073/pnas.1200678109 PMC3386081 22645367

[7] S. Vangaveti , T. Vreven , Y. Zhang , and Z. Weng , “Integrating Ab Initio and Template‐Based Algorithms for Protein‐Protein Complex Structure Prediction,” Bioinformatics 36, no. 3 (2020): 751–757. 10.1093/bioinformatics/btz623 PMC7523679 31393558

[8] J. D. Guest , T. Vreven , J. Zhou , et al., “An Expanded Benchmark for Antibody‐Antigen Docking and Affinity Prediction Reveals Insights Into Antibody Recognition Determinants,” Structure 29, no. 6 (2021): 606–621 e605. 10.1016/j.str.2021.01.005 PMC8178184 33539768

[9] T. Vreven , I. H. Moal , A. Vangone , et al., “Updates to the Integrated Protein‐Protein Interaction Benchmarks: Docking Benchmark Version 5 and Affinity Benchmark Version 2,” Journal of Molecular Biology 427, no. 19 (2015): 3031–3041. 10.1016/j.jmb.2015.07.016 PMC4677049 26231283

[10] P. J. Kundrotas , I. Anishchenko , T. Dauzhenka , et al., “Dockground: A Comprehensive Data Resource for Modeling of Protein Complexes,” Protein Science 27, no. 1 (2018): 172–181. 10.1002/pro.3295 PMC5734278 28891124

[11] J. Janin , K. Henrick , J. Moult , et al., “CAPRI: A Critical Assessment of PRedicted Interactions,” Proteins 52, no. 1 (2003): 2–9. 10.1002/prot.10381 12784359

[12] K. Wiehe , B. Pierce , J. Mintseris , et al., “ZDOCK and RDOCK Performance in CAPRI Rounds 3, 4, and 5,” Proteins 60, no. 2 (2005): 207–213. 10.1002/prot.20559 15981263

[13] K. Wiehe , B. Pierce , W. W. Tong , H. Hwang , J. Mintseris , and Z. Weng , “The Performance of ZDOCK and ZRANK in Rounds 6‐11 of CAPRI,” Proteins 69, no. 4 (2007): 719–725. 10.1002/prot.21747 17803212

[14] H. Hwang , T. Vreven , B. G. Pierce , J. H. Hung , and Z. Weng , “Performance of ZDOCK and ZRANK in CAPRI Rounds 13‐19,” Proteins 78, no. 15 (2010): 3104–3110. 10.1002/prot.22764 PMC3936321 20936681

[15] T. Vreven , B. G. Pierce , H. Hwang , and Z. Weng , “Performance of ZDOCK in CAPRI Rounds 20‐26,” Proteins 81, no. 12 (2013): 2175–2182. 10.1002/prot.24432 PMC3975700 24123140

[16] T. Vreven , B. G. Pierce , T. M. Borrman , and Z. Weng , “Performance of ZDOCK and IRAD in CAPRI Rounds 28‐34,” Proteins 85, no. 3 (2017): 408–416. 10.1002/prot.25186 PMC5313317 27718275

[17] M. F. Lensink , G. Brysbaert , N. Nadzirin , et al., “Blind Prediction of Homo‐ and Hetero‐Protein Complexes: The CASP13‐CAPRI Experiment,” Proteins 87, no. 12 (2019): 1200–1221. 10.1002/prot.25838 PMC7274794 31612567

[18] M. F. Lensink , G. Brysbaert , T. Mauri , et al., “Prediction of Protein Assemblies, the Next Frontier: The CASP14‐CAPRI Experiment,” Proteins 89, no. 12 (2021): 1800–1823. 10.1002/prot.26222 PMC8616814 34453465

[19] M. F. Lensink , G. Brysbaert , N. Raouraoua , et al., “Impact of AlphaFold on Structure Prediction of Protein Complexes: The CASP15‐CAPRI Experiment,” Proteins 91, no. 12 (2023): 1658–1683. 10.1002/prot.26609 PMC10841881 37905971

[20] R. Evans , M. O'Neill , A. Pritzel , et al., “Protein Complex Prediction With AlphaFold‐Multimer,” bioRxiv (2021): 2021.10.04.463034.

[21] A. Sircar , E. T. Kim , and J. J. Gray , “RosettaAntibody: Antibody Variable Region Homology Modeling Server,” Nucleic Acids Research 37 (2009): W474–W479. 10.1093/nar/gkp387 PMC2703951 19458157

[22] B. G. Pierce , Y. Hourai , and Z. Weng , “Accelerating Protein Docking in ZDOCK Using an Advanced 3D Convolution Library,” PLoS One 6, no. 9 (2011): e24657. 10.1371/journal.pone.0024657 PMC3176283 21949741

[23] A. Sali and T. L. Blundell , “Comparative Protein Modelling by Satisfaction of Spatial Restraints,” Journal of Molecular Biology 234, no. 3 (1993): 779–815. 10.1006/jmbi.1993.1626 8254673

[24] I. Andre , P. Bradley , C. Wang , and D. Baker , “Prediction of the Structure of Symmetrical Protein Assemblies,” Proceedings of the National Academy of Sciences of the United States of America 104, no. 45 (2007): 17656–17661. 10.1073/pnas.0702626104 PMC2077069 17978193

[25] B. Pierce , W. Tong , and Z. Weng , “M‐ZDOCK: A Grid‐Based Approach for Cn Symmetric Multimer Docking,” Bioinformatics 21, no. 8 (2005): 1472–1478. 10.1093/bioinformatics/bti229 15613396

[26] A. Waterhouse , M. Bertoni , S. Bienert , et al., “SWISS‐MODEL: Homology Modelling of Protein Structures and Complexes,” Nucleic Acids Research 46, no. W1 (2018): W296–W303. 10.1093/nar/gky427 PMC6030848 29788355

[27] A. Stein and T. Kortemme , “Improvements to Robotics‐Inspired Conformational Sampling in Rosetta,” PLoS One 8, no. 5 (2013): e63090. 10.1371/journal.pone.0063090 PMC3660577 23704889

[28] J. Jumper , R. Evans , A. Pritzel , et al., “Highly Accurate Protein Structure Prediction With AlphaFold,” Nature 596, no. 7873 (2021): 583–589. 10.1038/s41586-021-03819-2 PMC8371605 34265844

[29] Y. Yan , H. Tao , J. He , and S. Y. Huang , “The HDOCK Server for Integrated Protein‐Protein Docking,” Nature Protocols 15, no. 5 (2020): 1829–1852. 10.1038/s41596-020-0312-x 32269383

[30] B. Wallner , “AFsample: Improving Multimer Prediction With AlphaFold Using Massive Sampling,” Bioinformatics 39, no. 9 (2023): btad573. 10.1093/bioinformatics/btad573 PMC10534052 37713472

[31] J. P. Rodrigues , M. Trellet , C. Schmitz , et al., “Clustering Biomolecular Complexes by Residue Contacts Similarity,” Proteins 80, no. 7 (2012): 1810–1817. 10.1002/prot.24078 22489062

[32] F. Khatib , S. Cooper , M. D. Tyka , et al., “Algorithm Discovery by Protein Folding Game Players,” Proceedings of the National Academy of Sciences of the United States of America 108, no. 47 (2011): 18949–18953. 10.1073/pnas.1115898108 PMC3223433 22065763

[33] T. Vreven , H. Hwang , and Z. Weng , “Integrating Atom‐Based and Residue‐Based Scoring Functions for Protein‐Protein Docking,” Protein Science 20, no. 9 (2011): 1576–1586. 10.1002/pro.687 PMC3190152 21739500

[34] B. Pierce and Z. Weng , “ZRANK: Reranking Protein Docking Predictions With an Optimized Energy Function,” Proteins 67, no. 4 (2007): 1078–1086. 10.1002/prot.21373 17373710

[35] B. Pierce and Z. Weng , “A Combination of Rescoring and Refinement Significantly Improves Protein Docking Performance,” Proteins 72, no. 1 (2008): 270–279. 10.1002/prot.21920 PMC2696687 18214977

[36] G. Kleiger , A. Saha , S. Lewis , B. Kuhlman , and R. J. Deshaies , “Rapid E2‐E3 Assembly and Disassembly Enable Processive Ubiquitylation of Cullin‐RING Ubiquitin Ligase Substrates,” Cell 139, no. 5 (2009): 957–968. 10.1016/j.cell.2009.10.030 PMC2804849 19945379

[37] P. B. Stranges and B. Kuhlman , “A Comparison of Successful and Failed Protein Interface Designs Highlights the Challenges of Designing Buried Hydrogen Bonds,” Protein Science 22, no. 1 (2013): 74–82. 10.1002/pro.2187 PMC3575862 23139141

[38] R. F. Alford , A. Leaver‐Fay , J. R. Jeliazkov , et al., “The Rosetta all‐Atom Energy Function for Macromolecular Modeling and Design,” Journal of Chemical Theory and Computation 13, no. 6 (2017): 3031–3048. 10.1021/acs.jctc.7b00125 PMC5717763 28430426

[39] F. Fanelli and S. Ferrari , “Prediction of MEF2A‐DNA Interface by Rigid Body Docking: A Tool for Fast Estimation of Protein Mutational Effects on DNA Binding,” Journal of Structural Biology 153, no. 3 (2006): 278–283. 10.1016/j.jsb.2005.12.002 16427316

[40] R. Yin and B. G. Pierce , “Evaluation of AlphaFold Antibody‐Antigen Modeling With Implications for Improving Predictive Accuracy,” Protein Science 33, no. 1 (2024): e4865. 10.1002/pro.4865 PMC10751731 38073135

[41] J. W. Beugelink , E. Sweep , B. J. C. Janssen , J. Snijder , and M. F. Pronker , “Structural Basis for Recognition of the FLAG‐Tag by Anti‐FLAG M2,” Journal of Molecular Biology 436, no. 16 (2024): 168649. 10.1016/j.jmb.2024.168649 38852931

[42] S. Basu and B. Wallner , “DockQ: A Quality Measure for Protein‐Protein Docking Models,” PLoS One 11, no. 8 (2016): e0161879. 10.1371/journal.pone.0161879 PMC4999177 27560519

[43] J. Abramson , J. Adler , J. Dunger , et al., “Accurate Structure Prediction of Biomolecular Interactions With AlphaFold 3,” Nature 630 (2024): 493–500. 10.1038/s41586-024-07487-w PMC11168924 38718835

[44] R. Yin , B. Y. Feng , A. Varshney , and B. G. Pierce , “Benchmarking AlphaFold for Protein Complex Modeling Reveals Accuracy Determinants,” Protein Science 31, no. 8 (2022): e4379. 10.1002/pro.4379 PMC9278006 35900023

[45] K. Olechnovic , L. Valancauskas , J. Dapkunas , and C. Venclovas , “Prediction of Protein Assemblies by Structure Sampling Followed by Interface‐Focused Scoring,” Proteins 91, no. 12 (2023): 1724–1733. 10.1002/prot.26569 37578163

